# Incorporating Machine Learning Techniques to Enhance Rodent Surveillance in Marginalized Urban Communities

**DOI:** 10.1002/ece3.72382

**Published:** 2025-10-30

**Authors:** Fabio Neves Souza, Adedayo Michael Awoniyi, Rodrigo Dalvit Carvalho da Silva, Nivison Nery, Maria Victoria Moraes Oliveira, Caio Graco Zeppelini, George Andre Pereira Thé, Kathryn Hacker, Max T. Eyre, Hernan Dario Argibay, Albert Ko, Federico Costa, Hussein Khalil

**Affiliations:** ^1^ Instituto de Saúde Coletiva Universidade Federal da Bahia Salvador BA Brazil; ^2^ Instituto Gonçalo Moniz Fundação Oswaldo Cruz Salvador Bahia Brazil; ^3^ Neurosurgery, School of Medicine Yale University New Haven Connecticut USA; ^4^ Departamento de Engenharia de Teleinformatica Universidade Federal do Ceara – CE Fortaleza Brazil; ^5^ Department of Epidemiology University of Michigan Ann Arbor Michigan USA; ^6^ Faculty of Infectious Tropical Diseases, Disease Control Department London School of Hygiene & Tropical Medicine London UK; ^7^ Department of Epidemiology of Microbial Diseases Yale School of Public Health New Haven Connecticut USA; ^8^ Lancaster University Lancaster Medical School Lancaster UK; ^9^ Department of Wildlife, Fish and Environmental Studies (VFM) Swedish University of Agricultural Sciencies (SLU) Uppsala Sweden

**Keywords:** machine learning, pest rodents, principal component analysis, rodent management, zoonoses control

## Abstract

Effective management of rodent pests necessitates efficient population surveillance. Many of the available methods currently used for estimating rodent populations are either costly or time‐intensive. Rodent trapping demands significant resources, while tracking plates (TP) require high technical expertise and weeks to months of dedicated effort to satisfactorily interpret the plates. Here, we propose integrating Machine Learning techniques to evaluate plates with signs of rodent marks and compare their accuracy with that of conventional human‐interpreted plates. We employed the Otsu method to transform plates from RGB color images to grayscale images, highlighting regions of interest. Subsequently, we applied a global threshold to create binary images, assigning values above a globally determined threshold as 1s and others as 0s. The original images were transformed into new versions with 25 small samples, highlighting regions of interest based on the binary images. We used dimensionality reduction methods to identify the fundamental structure of high‐dimensional data and determined the most important patterns of interest on the plates. Among the methods, Principal Component Analysis, Independent Component Analysis, and Legendre Moments methods were used to visualize patterns and conduct exploratory data analysis. The *k*‐nearest neighbors, a versatile and intuitive classification method relying on the similarity principle, predicted the feature vector of PCA, ICA, and LM ($L_{\mathrm{pq}}$) results. Ultimately, results from PCA and LM compared favorably against the conventional labur‐intensive manual method, thus proffering those in the field of disease ecology a better alternative for conducting timely and cost‐effective rodent surveillance to monitor rodent distribution hotspots during rodent management programs. We propose a novel approach that could significantly enhance the protocols of rodent surveillance programs, particularly in Low‐ and Middle‐Income Countries, where expertise in interpreting TPs may be limited to enhance rodent surveillance evaluation and timely rodent management while contributing to the indirect control of rodent‐borne zoonoses.

## Graphical abstract

Represent the in‐field design of the trackplates and analysis methods.

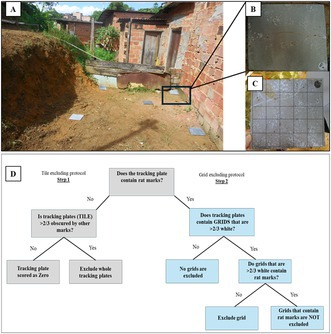

## 1 Introduction

Rodents are ubiquitous in both rural and urban environments, exerting a significant impact on global economies, with estimated annual agricultural and household losses reaching approximately US $22 billion (Belmain et al. 2014; Diagne et al. 2023). As global temperatures rise due to climate change, shifting environmental conditions are likely to alter rodent populations, amplifying their economic, ecological, and public health impacts (Costa, Porter, et al. 2014; Islam et al. 2021; Meerburg et al. 2009; Richardson et al. 2025). Increases in rodent abundance and geographic distribution may elevate the risk of zoonotic disease spillover, including bartonellosis (Zeppelini et al. 2023), Lassa fever (Olayemi et al. 2016), leptospirosis (Costa, Porter, et al. 2014), plague (Vall et al. 2020), salmonellosis (Falay et al. 2022), and toxoplasmosis (Johnson and Koshy 2020) among others. Additionally, rat sightings can impact mental health, often leading to disturbed sleep and psychological trauma among affected residents (Byers et al. 2019; Chelule and Mbentse 2021; de Klerk et al. 2016).

The agricultural, public health, and socioeconomic importance of rodent proliferation often necessitates the use of control measures, preferably non‐hazardous methods (Stuart et al. 2024) to mitigate rodent‐related damage, agricultural losses, the spread of rodent‐borne diseases, and the broader problem of rodent population infestation (Tobin and Fall 2004). To design and evaluate effective control strategies, we need a precise assessment of the target rodent species and population trends (Rahelinirina et al. 2021). Several methods are commonly used to evaluate rodent population proliferation in both agricultural and urban/rural environments. These include complaints or reports of rodent sightings (Awoniyi et al. 2021; Murray et al. 2018), exterior and interior inspection of households for active rodent signs (CDC 2006), rodent trapping (Woodman et al. 1996), assessment of rodent‐induced damage (Belmain et al. 2014), and tracking plates (TPs) (Hacker et al. 2016).

Many of these methods used for assessing rodent populations have inherent limitations. For example, complaints or reports of rodent sightings are subject to temporal biases, as they typically rely on observations made during the day or shortly before dusk, periods that do not coincide with the peak of rodent activity (Awoniyi et al. 2024). Rodent trapping, while suitable for both surveillance and control programs, is resource‐intensive and may be susceptible to rodent trap avoidance. For instance, Duron et al. (2020) estimated the cost of trapping during an intervention at approximately €38,000 for a single trapping session involving 10 persons working at least 4 h per day over 15 consecutive days to cover an average 200 ha‐sized plot in New Caledonia. This illustrates the financial and logistic burden that would be similarly expected during surveillance programs. Similarly, household exterior and interior inspections and TPs, a method validated as a proxy for rodent infestation and particularly useful in complex, marginalized urban terrains such as the marginalized urban communities in Salvador, Bahia, Brazil (Eyre et al. 2020; Hacker et al. 2016), also have notable setbacks. Although less capital‐intensive, TPs require technical expertise and considerable person‐time investment to process, as reading, scoring, and interpreting rodent markings can take weeks to months of dedicated work by highly trained staff. Nevertheless, unlike the conventional surveillance methods, TPs are generally not affected by trap avoidance behaviors. By passively recording rodent presence through footprints and other markings without the need to attract or confine the animals, TPs remain effective in environments where trap shyness undermines the efficiency of conventional techniques.

Therefore, it is imperative to develop a robust and reliable method for precisely evaluating rodent populations while overcoming the limitations associated with previous approaches and with advances the artificial intelligence showed opportunity tools in ecology studies. Here, we build on recent advances in Machine Learning (ML) techniques in ecological studies to integrate ML with TPs to evaluate rodent infestation, particularly in challenging urban terrains. Specifically, we aim to characterize the suitability of our novel method in accurately assessing rodent infestation by comparing the level of precision of the ML‐interpreted TPs with the conventional human‐interpreted plates. The results of this study should provide a prompt yet effective and reliable method for evaluating rodent infestation, which is also adaptable to other small mammal species in both rural and marginalized urban communities where their infestations have significant negative impacts on household properties, agriculture, public health, and economic productivity to guide informed small mammal population management.

## 2 Methods

### 2.1 Study Area

The tracking plate (TP) data utilized here was obtained from studies conducted in Pau da Lima (13°32′53.47″ S; 38°43′51.10″ W) between March and June 2015. Pau da Lima, situated in the outskirts of Salvador City, Bahia, Brazil, consists of a series of valleys, spanning an area of 0.17 km^2^, with approximately 128,997 inhabitants residing in this low‐income urban community (IBGE 2010). It features a subtropical climate, maintaining a relatively constant temperature throughout the year, but with varying rainfall across seasons. The rainy season, from April to July, typically experiences a mean of 272.2 mm/month, while the dry season from September to December sees a reduced mean rainfall of 124.2 mm/month. Pau da Lima is characterized by inappropriate solid waste management practices, an unsatisfactory sanitation system evidenced by open sewers and inadequate housing facilities (> 80% are squatters), contributing to a history of household rodent infestation (Costa, Ribeiro, et al. 2014).

### 2.2 Tracking Plates

#### 2.2.1 Positioning of the Tracking Plates (TPs)

**FIGURE 1:**
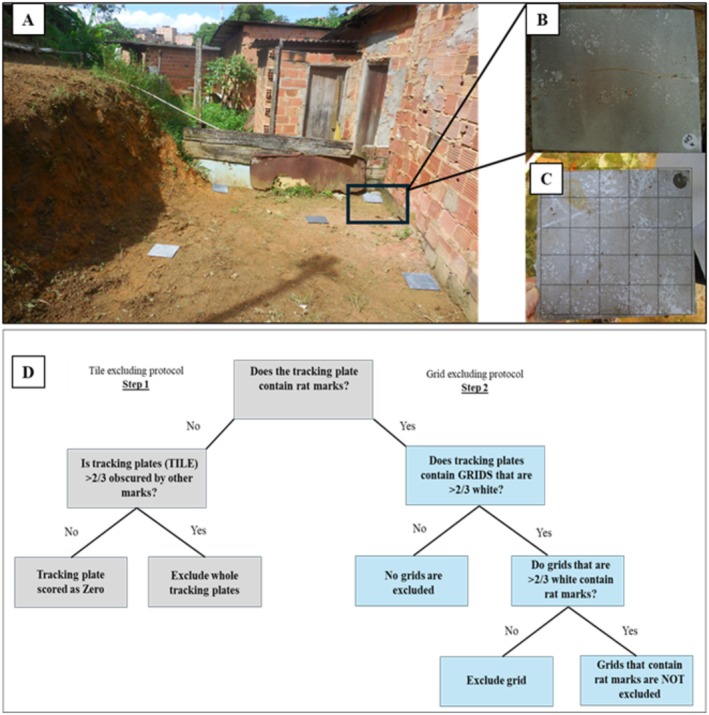
(A) Illustration of TP placement in the field, (B) an example of a TP with rodent marks, (C) illustration of a TP with rodent marks overlaid on a 5 by 5 grid for subsequent reading/scoring, and (D) schematic step‐by‐step analysis procedure of TP.

For this study, we randomly selected 4200 TPs positioned in 420 sampling points across Pau da Lima. Of these, 179 were lost or removed from the sampling points, resulting in a final count of 4021 TPs. Sixty‐seven tracking plates were deployed at 14 randomly selected locations within the study area. The procedure for placing and positioning the TPs has been extensively discussed by Hacker et al. (2016). Briefly, at each sampling point, we positioned five 0.2 × 0.2 m acetate polyvinyl plates (TPs) preferably in an X‐pattern (see Figure 1A), maintaining an approximately 1 m distance between plates. Specifically, after positioning the central plate, the others were positioned within a 1 m radius buffer (Figure 1A). Before TPs positioning in the sampling area, they were painted with weather‐resistant lampblack using a paint roller. Briefly, the lampblack is prepared by dissolving 10 g of lampblack powder in 200 mL of 70% ethanol in a tightly sealed container, followed by thorough mixing and allowing the solution to settle for at least 24 h before use. Once applied, the painted TPs dry within 5 min, enabling clear detection of various types of marks left by rodents such as paw prints, tail marks, and scratches (Figure 1B,C). We strategically placed TPs along natural barriers, but not in open areas to minimize attention from passers‐by.

We placed TPs at selected sampling points for a minimum of two consecutive days. To document information about each TP, we examined each painted TP the following dawn for signs of possible rodent marks, including the presence of scratches or marks on the TPs (potential positive signs of rodent activities—though not necessarily indicative of rodent activities) and its status (i.e., if the TP was moved or lost). All TPs found intact were labeled, indicating the location, TP number, and survey date. Labeled TPs were photographed and uploaded onto an online data management platform (REDCap), using the unique TP label for subsequent review (Wright 2016). All TPs were repainted and repositioned in the same sampling location as the previous day. To adjust for potential confounding due to rainfall, considering rodents may exhibit reduced activity during periods of intense rainfall, leading to reduced rodent marks and potentially rendering the TPs unreadable, we repeated the experiment whenever more than 10% of the TPs were unreadable due to intense rainfall until we achieved two consecutive days of readable TPs.

### 2.3 Scoring and Analysis of Tracking Plates (TPs)

Following TPs collection from the field, they were scored using the procedure previously described by Hacker et al. (2016) and Eyre et al. (2020). Specifically, all digital images of the TPs were overlaid on a 5 × 5 grid (Figure 1C). Two independent examiners scored the overlaid TPs by reviewing specific signs of rat markings within the 5 × 5 grid. Discordant scores of more than three‐cell difference between examiners were reviewed to obtain a consensus. Overall TP scores were calculated using the number of positive cells among each of the positioned TPs during the sampling period (i.e., the mean score of all five TPs). Individual TPs were excluded if the observed markings covering the grid precluded rodent‐specific markings (Figure 1D). The principal outcome of each TP was calculated as the proportion of cells with rodent marks (i.e., the number of cells positive for rodent markings divided by 25, which corresponds to the total number of cells on each TP). All TP marks determined to be non‐rodent marks were considered “negative.”

### 2.4 Machine Learning Study Procedure

#### 2.4.1 Pre‐Processing

**FIGURE 2:**
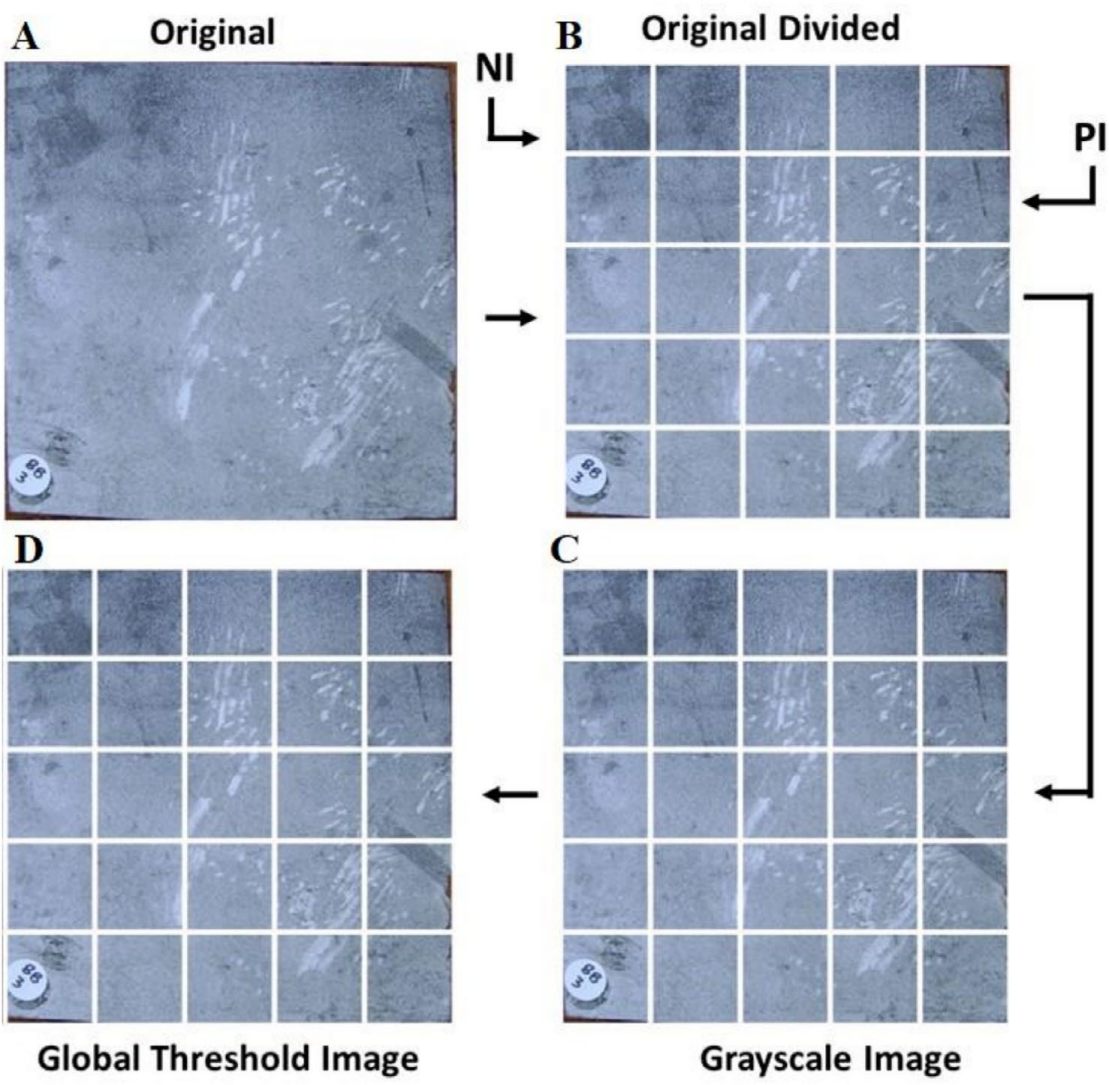
Pre‐processing workflow. (A) Original RGB plate image; (B) 25 small sub‐images sampled from the original plate; (C) grayscale conversion of the image; (D) global threshold applied, determined from the first image, producing binary images. The resulting 25‐cell sub‐images were used as input for the machine learning model.

In total, 67 TPs were then transformed from RGB (red, green, and blue) to grayscale. To highlight regions of interest, a global threshold, determined from the mean intensity of the first image, was applied to all images. Pixels with intensity above this threshold were set to 1, and those below were set to 0, producing binary images (Figure 2).

From these 67 TPs, 44 were identified as positive images (PI), images that showed signs of rodent marks, and 23 as images with non‐rodent marks or negative images (NI). These TPs were divided into 25 sub‐samples as a preprocessing step to highlight localized regions of interest and to augment the dataset for training the ML models, since subtle rodent marks often occupy only small portions of a plate. This subdivision does not redefine the biological unit of measurement, which remains the entire plate. For analysis, sub‐sample classifications can be aggregated back to the plate level: if one or more sub‐samples are identified as positive, the plate is considered active (positive); if no sub‐samples are positive, the plate is considered not active (negative). Thus, while subdivision improves sensitivity and provides sufficient training data, rodent activity is ultimately assessed at the plate level. From the 44 PI and 23 NI, 1100 (PI) and 575 (NI) subsets of images were generated (25 samples per image), respectively. To identify rodent marks from the subset of 1100 images, four experts evaluated each image individually, and images that had at least three votes for positive were selected to create a positive dataset. From the 1100 initial positive sub‐samples, 654 were labeled as containing rodent marks. The remaining 446 without marks were combined with the 575 negative sub‐samples, yielding a dataset of 654 PI and 1021 NI. Figure 2 displays sub‐samples of PI and NI.

#### 2.4.2 Feature Extraction

Feature extraction tackles the task of identifying the most succinct and informative feature set while reducing the size of the original data, optimizing both data storage and processing efficiency (Guyon et al. 2008). It improves machine learning classification methods by reducing the number of overfittings. Various methods, including principal component analysis, independent component analysis, t‐distributed stochastic neighbor embedding, and moment functions, can be employed for feature extraction.

#### 2.4.3 Principal Component Analysis

Principal Component Analysis (PCA), introduced by Pearson (1901) and developed by Hotelling (1933), is a fundamental technique for data analysis and dimensionality reduction. PCA identifies key patterns and correlations in high‐dimensional data by transforming the original variables into uncorrelated Principal Components (PCs) that capture the greatest variance. This simplification retains essential information while reducing complexity. Widely used in fields such as signal processing, machine learning, and statistics, PCA is an effective tool for noise reduction, pattern detection, and enhancing the interpretation of multivariate data.

#### 2.4.4 Independent Component Analysis

The principle of Independent Component Analysis (ICA), a statistical signal processing method for linearly decomposing a random vector into components that strive for maximum independence (Hyvärinen et al. 2001) was employed in the classification of TP images under test. The fundamental principle of ICA involves observations of random variables $\left\{x_{1}\left(t\right),x_{2}\left(t\right),\ldots ,x_{n}\left(t\right)\right\}$, presumed to originate from a linear mixture of independent components $\left\{s_{1}\left(t\right),s_{2}\left(t\right),\ldots ,s_{n}\left(t\right)\right\}$, according to

(1) $$x={\left(x_{1}\left(t\right),x_{2}\left(t\right),\ldots ,x_{n}\left(t\right)\right)}^{T}=A{\left(s_{1}\left(t\right),s_{2}\left(t\right),\ldots ,s_{n}\left(t\right)\right)}^{T}=\mathrm{As}$$

where A is the unknown mixture matrix. In the context of feature extraction using ICA, the columns of $A_{\text{train}}$ comprise the primary feature vectors extracted from the training images. These vectors serve as input for the classifier, combined with the mixture matrix of the image under test, $A_{\text{test}}$.

**FIGURE 3:**
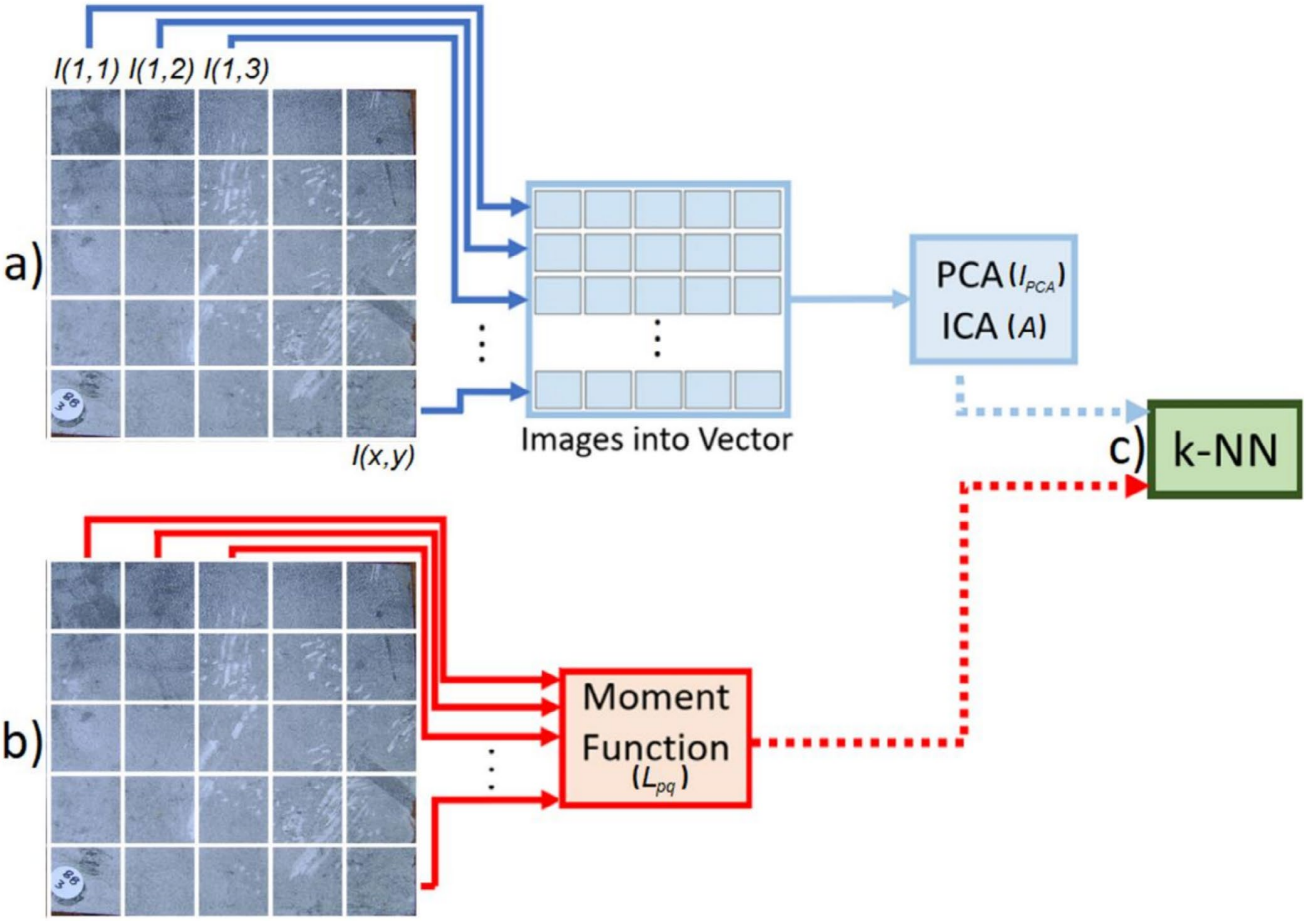
The feature extraction stage involves several steps. (a) The 25 small samples are vectorized and then stacked of each other. Then, this combined dataset is input into PCA and ICA for dimensionality reduction. (b) Images are directly inputted into the Moment Functions for processing. (c) Feature vectors generated from all applied methods are fed into *k*‐NN algorithm for classification purposes. ICA, Independent Component Analysis; *k*‐NN, *k*‐nearest neighbors; L, Legendre Moments; PCA, Principal Components Analysis.

In this study, the images $x_{n}$ (or $I\left(x,y\right)$) are vectorized ($I_{v}$) and stacked. The $I_{v}$ are processed by the PCA algorithm to compute their respective principal components (PCs) representations, which are used to project $I_{v}$ onto the subspace spanned by the selected PCs. This is done by multiplying $I_{v}$ by the selected PCs, $I_{\mathrm{PCA}}=I_{v}*\mathrm{PCs}$. For the ICA method, $I_{v}$ is fed into the FastICA algorithm (Hyvärinen and Oja 2000), which calculates A and s. The matrix A, analogous to the $I_{\mathrm{PCA}}$ in PCA, is used as the ICA feature descriptor for each image, as illustrated in Figure 3a.

#### 2.4.5 Moment Functions

The moment methods provide an effective manner to extract rotation‐invariant features from images (Teague 1980). The image moment can be described mathematically as the inner product of the basis function $R_{\mathrm{ns}}\left(r\right)$ and the image function $f\left(r,\theta \right)$ (2D image defined in polar coordinate system). The image moment sets can be directly interpreted geometrically as the projection of $f\left(r,\theta \right)$ onto a subspace formed by a set of basic functions $R_{\mathrm{ns}}\left(r\right)$. Finding a collection of unique basis functions with advantageous qualities can be challenging given that there exist several basis function sets.

A simple moment function that can be easily implemented is the Legendre Moments (LM) which was introduced by Teague (1980). It is produced based on the recurrence relation of Legendre polynomial of order p (Chong et al. 2004), and is defined as

(2) $$\mathrm{Pp}\left(x\right)=\frac{\left(2p-1\right)\mathrm{xPp}-1\left(x\right)-\left(p-1\right)\mathrm{Pp}-2\left(x\right)}{p}$$

where $P_{0}\left(x\right)=1$, $P_{1}\left(x\right)=x$, and p>1. Since the region of definition of Legendre polynomial is the interior of $\left[-1,1\right]$, a square image $I\left(x,y\right)$ of N×N pixels with image pixel coordinates (x, y), typically ranging from $\left[0,N-1\right]$, is scaled in the region −1<x,y<1. The discrete form of the LM of order $\left(p+q\right)$ for an image $I\left(x,y\right)$ can be expressed as

(3) $$\mathrm{Lpq}=\mathrm{\lambda pq}\sum_{x=0}^{N-1}\sum_{y=0}^{N-1}\mathrm{Pp}\left(\hat{x}\right)\mathrm{Pq}\left(\hat{y}\right)I\left(x,y\right)$$

where the normalizing constant is

(4) $$\mathrm{\lambda pq}=\frac{\left(2p+1\right)\left(2q+1\right)}{N^{2}}$$

$\hat{x}$ and $\hat{y}$ denote the normalized pixel coordinates in the range $\left[-1,1\right]$, which are given by

(5) $$\hat{x}=\frac{2i}{N-1}-1,\;\hat{y}=\frac{2j}{N-1}-1$$

Images $I\left(x,y\right)$ are input into the LM algorithm, which computes their corresponding moments using Legendre polynomials $L_{\mathrm{pq}}$. Unlike PCA and ICA techniques, LM do not require vectorization; instead, the images can be directly used to calculate the $L_{\mathrm{pq}}$, as seen in Figure 3b.

#### 2.4.6 Classifier

Classifiers are the fundamental components of machine learning and pattern recognition. They are essential for classifying and generating predictions from newly received data.

#### 2.4.7 *k*‐Nearest Neighbors

A versatile and intuitive classification method, *k*‐Nearest Neighbors (*k*‐NN) relies on the principle of similarity to classify data points based on their proximity to neighboring instances. The technique, which was initially presented by Fix and Hodges (1951) and then refined by Cover and Hart (1967), uses a feature space to classify data points based on how close they are to known labeled data points.

Fundamentally, *k*‐NN uses the labels of its closest neighbors when determining the grouping of a new data point. The “*k*” in *k*‐NN refers to the number of neighbors that are considered while classifying. The algorithm searches the *k* nearest data points depending on a chosen distance metric, such as Euclidean distance, to label a new data point. For each of these *k* neighbors, the most prevalent class becomes the predicted class for the new data point. The output derived from PCA ($I_{\mathrm{PCA}}$), ICA (A), and LM ($L_{\mathrm{pq}}$) serve as the input for the *k*‐NN algorithm, which computes the distances to identify the closest neighbors (Figure 3c). Table 1 displays the feature vector size for each method, along with the corresponding extraction time per sample processed on an Intel i7‐1255U (4.7 GHz), 16GB of RAM, and solid‐state drive (SSD) laptop. The codes were implemented in MATLAB 2023b.

**TABLE 1:** Total of components and extraction time of the feature vectors.

	Total of components (per sample)	Extraction time (s) (per sample)
ICA	100	1.0508
LM	100	0.4532
PCA	100	0.1843

## 3 Results

### 3.1 Outcomes of Conventional Tracking Plate Analysis

In total, we placed 6645 units of TPs, and recovered 5746 units (87.79%), out of which 3181 (55%) had marks from both rodent and non‐target species. Following the procedure to examine only TPs with at least two‐thirds marks (Connors et al. 2005), two independently trained evaluators examined 1675 TP units that satisfied this condition, corresponding to 526 (31.4%) TP units with rodent marks, notably with 80% agreement in the results of the two trained independent evaluators using Hacker et al. (2016) previously validated protocol.

The process of sorting TPs with both rodent and non‐rodent marks alone took weeks of dedicated evaluation by two well‐experienced evaluators. Additionally, identifying and scoring only TPs with rodent marks (in this case, 526 of 1675) took another week of evaluation, eventually yielding 80% concordance results from the two experienced evaluators.

### 3.2 Outcomes of Machine Learning Tracking Plate Analysis

**FIGURE 4:**
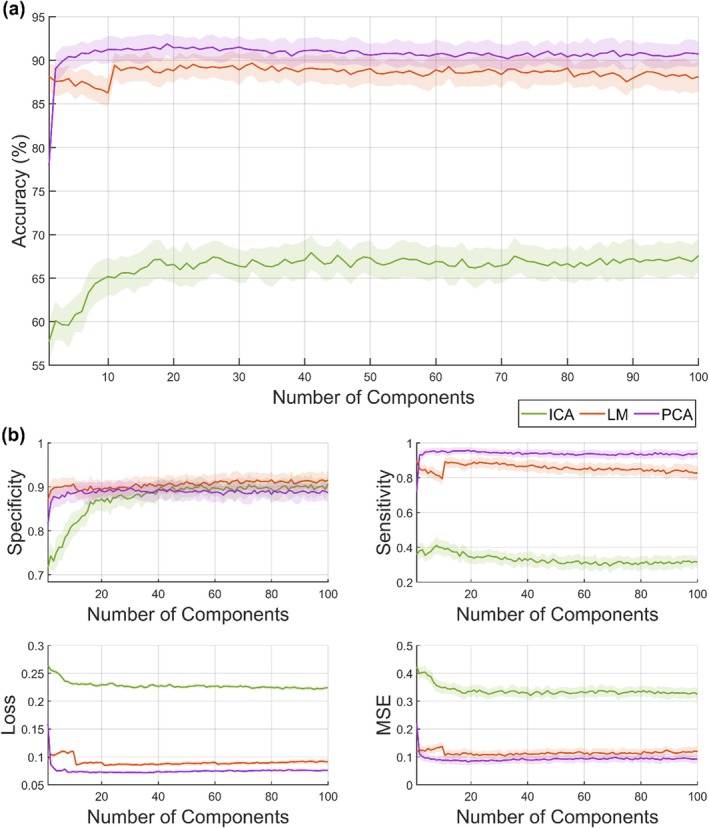
(a) Accuracy per feature vector size for all feature vectors; (b) specificity, sensitivity, cross‐entropy loss, and mean‐squared error when the number of components varies. ICA, Independent Component Analysis; LM, Legendre Moments; PCA, Principal Components Analysis.

To evaluate the performance of our ML model, we employed a repeated partitioning strategy. The dataset consisted of 67 plates → 654 positive images (PI), 1021 negative images (NI) of rodent marks and was partitioned 50 times. In each partition, the images were randomly split such that 80% of the PIs and 80% of the NIs were used for training while the remaining 20% of each class (PI and NI) were reserved for testing (data not shown). Feature vectors extracted from the images were then presented to a *k*‐nearest neighbor (*k*‐NN) classifier using the Euclidean distance metric, considering the 5 nearest neighbors for classification. This procedure was repeated across all iterations (50 times) to determine the optimal feature vector size. Figure 4 summarizes the overall classification performance. To evaluate model performance, we plotted accuracy as a function of feature vector size (Figure 4a) and reported additional metrics, including specificity, sensitivity, cross‐entropy loss, and mean‐squared error (MSE) (Figure 4b). Accuracy was emphasized as the primary metric since it reflects the overall agreement between predictions and ground truth. Sensitivity and specificity provided complementary insight into false negative and false positive errors, respectively, while cross‐entropy loss and MSE quantified prediction confidence and error magnitude, offering a more comprehensive assessment of model performance.

Results from Figure 4 and Table 1 indicate that the ML satisfactorily, especially PCA and LM identified and classified different marks on the TPs, particularly doing well for rodent identification with higher precision (bar ICA) and significantly shorter period (1 day) compared to the conventional method, which is rigorous and laborious and requires over 2 weeks of dedicated evaluation by two highly experienced evaluators.

**FIGURE 5:**
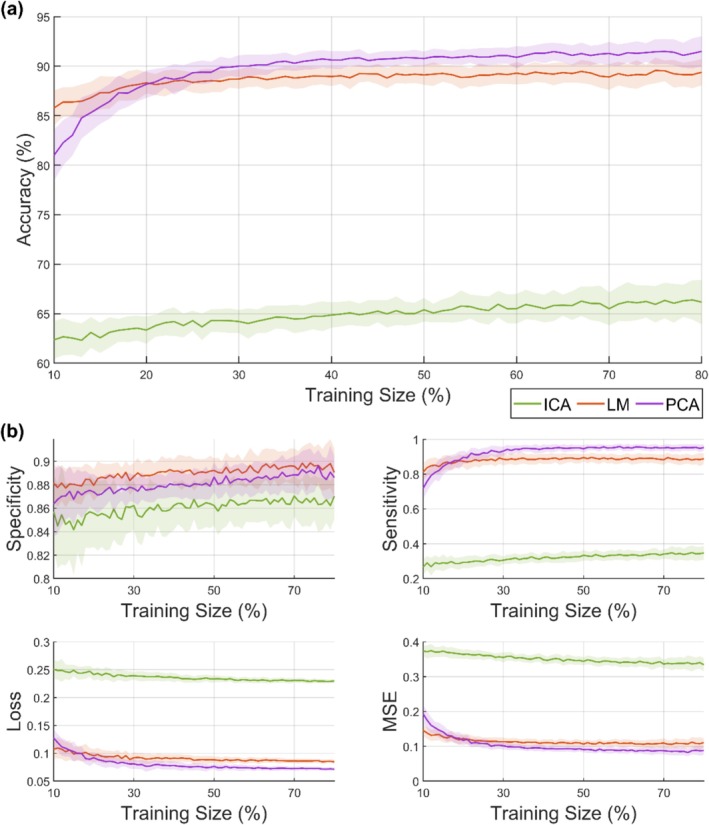
(a) Accuracy per descriptor when training set is varied; (b) Classification metrics when the training set varies from 10% to 80%. ICA, Independent Component Analysis; LM, Legendre Moments; PCA, Principal Components Analysis.

Figure 5 shows the efficacy of the feature extractors with varying training set sizes (10%–80%), keeping the remaining data for testing and using the same classification parameters as in the previous experiment. It presents the classification metrics, including sensitivity, specificity, cross‐entropy loss, and mean squared error (MSE), along with the Area Under the ROC (Receiver Operating Characteristic) curve (AUC) for each descriptor when 80% of the dataset is used for training.

## 4 Discussion

Incorporating machine learning in rodent surveillance could enhance efficiency and accuracy compared to conventional, labor‐intensive human analysis. In this study, two independent evaluators required over 2 weeks to classify 1675 plates, whereas the ML pipeline completed the same task in less than 1 day on a standard computer (Intel i7‐1255U, 16 GB RAM), yielding satisfactory results. Beyond reducing processing time, ML has the potential to reduce human error, increase confidence in the results, and detect subtle rodent marks that may be difficult for the human eye to spot, thereby improving the effectiveness of rodent surveillance programs.

Here, the TP‐rodent evaluation method performed well in detecting rodent infestations in the marginalized communities. Although TP‐rodent sampling is less expensive, simplified, easy to apply, and community friendly compared to other methods such as rodent trapping, exterior and interior rodent evaluation, the evaluation of the plates requires dedicated efforts and expertise. The intensity of rodent marks and the binary index of presence/absence of specific rodent marks on the TPs may be subjected to human error during analyses, as they often exhibit nonspecific markings that may be confusing to novice evaluators during analysis, thereby complicating the results of the final analysis.

Although the application of ML in ecology remains limited, it has significantly enhanced rodent behavioral analysis and the spatiotemporal dynamics evaluation of animal motion in ecology and neuroscience research, providing higher consistency than manual classification (Isik and Unal 2023; Luxem et al. 2022). Similarly, ML has shown potential to support clinical diagnostics and in some cases may complement or enhance conventional physician assessments (Farzaneh et al. 2023). In this study, in terms of processing time, Table 1 indicates that the extraction processing time for the feature extractors is different. The higher requirement of ICA compared to other methods during the extraction process might be attributed to the higher computational demand resulting from the numerous summations in the equations, often necessitating iterative loops at the computational level.

In applying ML to rodent surveillance analysis, the feature extractors based on PCA and moment functions (LM) tend to preserve crucial image information within a smaller set of components compared to ICA, as we can see in Figure 4. Their ability to handle inherent image invariances plays a significant role in achieving high accuracy with fewer components. For instance, PCA achieved 91.80% accuracy using 22 components compared to 89.05% for LM and 66.03% for ICA, which obtained the lowest overall accuracy rate. When aggregated at the plate level, ML classifications (PCA + *k*‐NN) of active versus inactive matched human scoring in 91.80% of cases. The observed low accuracies were anticipated and could be attributed to the inherent impact of image invariances, such as rotation, translation, and scaling, aligned with discussions outlined in other research papers (Ridgeway 2016; Silva et al. 2015). While PCA required 22 components to achieve peak accuracy (91.80%), classification metrics plateaued after 20 components, indicating stability rather than over‐complexity. The higher training proportion (50%–80%) was necessitated by the modest dataset size. In larger datasets, fewer training iterations would be expected. Thus, although training requirements were relatively high in this pilot study, the approach is scalable and generalizable with expanded data.

Figure 4 compares key classification metrics (sensitivity, specificity, cross‐entropy loss, and mean square error) for the ICA, PCA, and LM models. ICA required over 35 components to match the specificity of PCA and LM. With 22 components, ICA's sensitivity (~0.34) was much lower than PCA (~0.94) and LM (~0.88). Cross‐entropy loss for ICA (~0.228) was higher than PCA (~0.072) and LM (~0.086), indicating poorer prediction alignment. MSE was also higher for ICA (~0.319) compared to PCA (~0.080) and LM (~0.115). PCA and LM outperformed ICA in this assessment.

A posterior analysis was conducted with the number of components fixed at 22, while varying the training size from 10% to 80% to determine the optimal training size for achieving high accuracy. As shown in Figure 5, LM initially achieved the highest accuracy up to a training size of 20%, after which it was surpassed by PCA. LM reached an accuracy plateau of 88.71% when the training size was 27%. In contrast, ICA continued to improve its accuracy as the training size increased but remained low, even with 80% of the data used for training, achieving an accuracy of only 67.80%. PCA's accuracy steadily increased as the training size grew, eventually plateauing at 91.00% when 59% of the training set was used.

Figure 5 presents a similar scenario to Figure 4, with PCA and LM outperforming ICA in classification metrics. PCA and LM both showed improvements in specificity, increasing from ~0.87 to ~0.89 for PCA and from ~0.88 to ~0.895 for LM, while ICA's specificity only improved from ~0.85 to ~0.87. Sensitivity saw a substantial jump for PCA, rising from ~0.73 to ~0.95, and a moderate increase for LM, from ~0.82 to ~0.88. However, ICA's sensitivity remained significantly lower, increasing only slightly from ~0.275 to ~0.35. In terms of losses, PCA achieved the lowest cross‐entropy loss at ~0.07, followed by LM at ~0.09, while ICA had a ~0.23 loss. The mean square error (MSE) was also lowest for PCA (~0.095), with LM at ~0.12 and ICA lagging behind with a high MSE of ~0.33.

**FIGURE 6:**
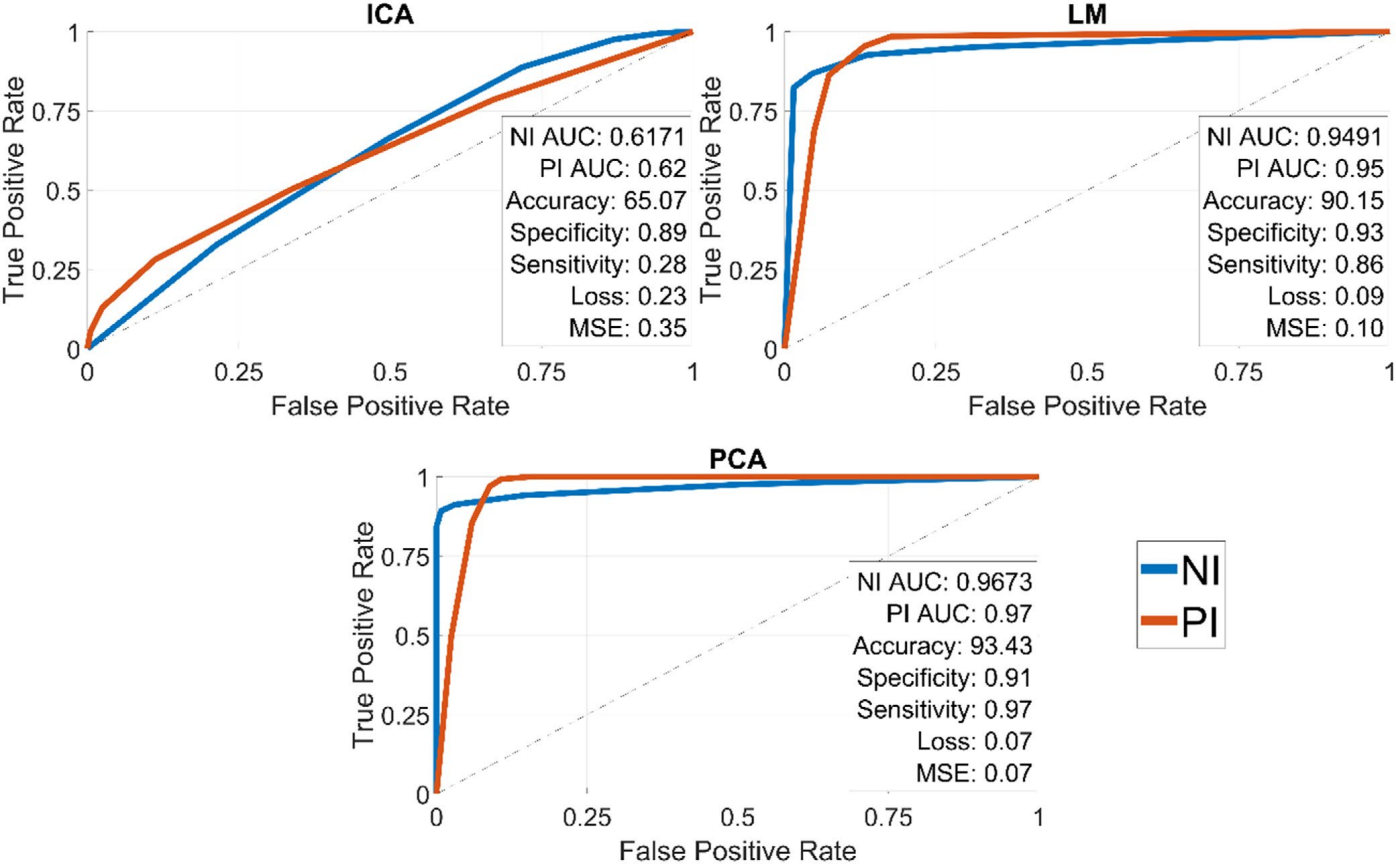
Area under the ROC curve (AUC) of all feature extractors when using a feature vector size of 22% and 80% of the data for training. ICA, Independent Component Analysis; LM, Legendre Moments; NI, negative images for rodent mark; PCA, Principal Components Analysis; PI, positive images.

Figure 6 presents the area under the curve (AUC) values for each model, offering insight into the quality of their predictions. The PCA and LM models achieved high AUC scores of 0.9343 and 0.9015, respectively, indicating strong abilities to distinguish between positive and negative classes. In contrast, the ICA model recorded a much lower AUC of 0.6507, reflecting a limited capacity to effectively differentiate between the two classes.

To our knowledge, this is the first paper presenting a method applied to rodent surveillance with ML. Another noteworthy study (Hopkins et al. 2024) demonstrates the use of camera traps enhanced with machine learning for rodent detection. Their ML model, using the YoloV5x, achieved high precision and recall (mean average precision of 0.941 over all seven rodent classes), indicating precise species identification from camera trap images. The findings of Hopkins et al. (2024) underscore the efficiency, practicality, and sustainability of combining camera traps with ML technology for ecological monitoring, highlighting its superior detection rates and reduced labor demand.

The importance of machine learning in this context cannot be overstated. Advanced ML algorithms enable automatic and accurate identification of rodent species from large datasets of camera trap images, enhancing both detection efficiency and data quality. By automating the identification process, ML reduces human labor and potential biases, ensuring consistent and reliable monitoring efforts. This technological advancement represents a significant step forward in wildlife conservation and ecological studies, as it allows for more effective and comprehensive monitoring of biodiversity.

Additionally, although TPs allow for the assessment of rodent presence based on the quantity of marks left by rodents, these can be challenging to identify and analyze using conventional methods; the integration of ML enhances these traditional approaches. By training algorithms to automatically identify these subtle details, which are difficult to detect manually, this approach represents a significant advancement in rodent population ecology and surveillance techniques. Additionally, the integration of ML and the conventional methods should enable satisfactory generalization of results and improve swift analysis of results, as a result reducing the limitations mentioned above regarding time and human errors in the identification and quantification of rodent marks on TPs.

A notable limitation of our current approach is that neither the conventional nor ML can identify the specific rodent species present during surveillance, as the TP evaluation is inherently non‐species specific. Consequently, a complementary surveillance method is required when species‐level identification is necessary. Going beyond simple presence/absence detection could be particularly valuable for rodent management programs, given that morphological differences among rodent species may differentially influence their population dynamics and management strategies (Keesing and Ostfeld 2024). Addressing this challenge remains an important objective for our subsequent studies.

## 5 Conclusion

The results of this study demonstrate the promising potential of incorporating ML into rodent surveillance. The superior performance of PCA and LM, with a higher degree of accuracy compared to the conventional labor‐intensive methods, highlights ML's ability to reduce human error during TP analysis. We present a novel approach that could significantly enhance the protocols of rodent surveillance programs. This method is particularly valuable for small mammal surveillance in LMICs, where expertise in interpreting TPs may be limited. Similarly, integrating ML into rodent surveillance should facilitate the prompt evaluation of rodent proliferation, enabling subsequent timely rodent population management, while also contributing to the indirect control of rodent‐borne zoonoses in marginalized urban communities.

## Author Contributions

**Fabio Neves Souza:** conceptualization (lead), data curation (lead), formal analysis (equal), project administration (lead), visualization (lead), writing – original draft (lead), writing – review and editing (lead). **Adedayo Michael Awoniyi:** conceptualization (lead), data curation (equal), formal analysis (lead), project administration (equal), visualization (equal), writing – original draft (lead), writing – review and editing (lead). **Rodrigo Dalvit Carvalho da Silva:** conceptualization (equal), data curation (lead), formal analysis (lead), methodology (lead), software (lead), visualization (lead), writing – original draft (lead), writing – review and editing (lead). **Nivison Nery Jr:** conceptualization (equal), project administration (equal), writing – original draft (equal), writing – review and editing (equal). **Maria Victoria Moraes Oliveira:** formal analysis (supporting), writing – review and editing (supporting). **Caio Graco Zeppelini:** project administration (supporting), writing – review and editing (equal). **George Andre Pereira Thé:** formal analysis (equal), writing – review and editing (equal). **Kathryn Hacker:** data curation (equal), project administration (supporting), writing – review and editing (equal). **Max T. Eyre:** project administration (equal), writing – review and editing (equal). **Hernan Dario Argibay:** project administration (equal), writing – review and editing (equal). **Albert Ko:** funding acquisition (equal), project administration (equal), writing – review and editing (equal). **Federico Costa:** funding acquisition (lead), project administration (lead), writing – review and editing (equal). **Hussein Khalil:** funding acquisition (lead), project administration (lead), writing – original draft (equal), writing – review and editing (equal).

## Disclosure

The authors have nothing to report.

## Conflicts of Interest

The authors declare no conflicts of interest.

## Data Availability

Models and full R code for simulating data study are available via https://doi.org/10.5281/zenodo.17256286.
